# Air Pollution and the Brain: A Harmonized Analysis of Four Cohorts With the Harmonized Cognitive Assessment Protocol

**DOI:** 10.1093/geroni/igaf122.046

**Published:** 2025-12-31

**Authors:** Boya Zhang

**Affiliations:** Harvard T.H. Chan School of Public Health, Boston, Massachusetts, United States

## Abstract

Growing evidence suggests that air pollution may have significant impacts on aging and cognitive health. This study investigates the cross-sectional associations of long-term exposure to total and source-specific fine particulate matter (PM2.5) with harmonized cognitive function measures across four countries. We included participants who had completed the Harmonized Cognitive Assessment Protocol battery of cognitive tests from Health and Retirement Study (HRS), a US nationally representative study, and its sister studies in England, Chile, and India. We linked modeled concentrations of total PM2.5 and PM2.5 from 19 emission sources to participants’ residential addresses over the 10 years preceding the cognitive assessment. To examine associations with cognitive function, we employed weighted generalized linear models adjusted for individual- and area-level confounders. The 10-year average total PM2.5 concentrations were ranging from 9.2±1.9 μg/m3 in the US to 56.5±25.9 μg/m3 in India. Although overall associations between total PM2.5 and cognitive function were modest across all countries, we identified universal and unique sources across countries. Specifically, higher wildfire-related PM2.5 was associated with poorer cognitive function in the US and India, while higher agriculture-related PM2.5 was linked to poor cognitive function in England and Chile, particularly among those living in the non-urban areas. Associations with residential PM2.5 were only observed for India. Our findings highlight the need for cross-nation studies to resolve air pollution issues from a global perspective.

